# Using Ambient Assisted Living to Monitor Older Adults With Alzheimer Disease: Single-Case Study to Validate the Monitoring Report

**DOI:** 10.2196/20215

**Published:** 2020-11-13

**Authors:** Maxime Lussier, Aline Aboujaoudé, Mélanie Couture, Maxim Moreau, Catherine Laliberté, Sylvain Giroux, Hélène Pigot, Sébastien Gaboury, Kévin Bouchard, Patricia Belchior, Carolina Bottari, Guy Paré, Charles Consel, Nathalie Bier

**Affiliations:** 1 Research Center of Institut universitaire de gériatrie de Montréal Integrated Health and Social Services University Network for South-Central Montreal Montreal, QC Canada; 2 School of Rehabilitation Faculty of Medicine Université de Montréal Montreal, QC Canada; 3 Integrated Health and Social Services University Network for West-Central Montreal Université de Sherbrooke Sherbrooke, QC Canada; 4 Department of Psychology Université de Sherbrooke Sherbrooke, QC Canada; 5 Research Chair in Digital Health High Commercial Studies of Montreal Montreal, QC Canada; 6 Faculty of Sciences and Faculty of Medicine and Health Sciences Université de Sherbrooke Sherbrooke, QC Canada; 7 Department of Mathematics and Computer Science Université du Québec à Chicoutimi Chicoutimi, QC Canada; 8 School of Physical and Occupational Therapy McGill University Montreal, QC Canada; 9 Bordeaux Institute of Technology & Inria Bordeaux France

**Keywords:** activities of daily living, aging, Alzheimer disease, ambient assisted living, health care, technology assessment, health, remote sensing technology

## Abstract

**Background:**

Many older adults choose to live independently in their homes for as long as possible, despite psychosocial and medical conditions that compromise their independence in daily living and safety. Faced with unprecedented challenges in allocating resources, home care administrators are increasingly open to using monitoring technologies known as ambient assisted living (AAL) to better support care recipients. To be effective, these technologies should be able to report clinically relevant changes to support decision making at an individual level.

**Objective:**

The aim of this study is to examine the concurrent validity of AAL monitoring reports and information gathered by care professionals using triangulation.

**Methods:**

This longitudinal single-case study spans over 490 days of monitoring a 90-year-old woman with Alzheimer disease receiving support from local health care services. A clinical nurse in charge of her health and social care was interviewed 3 times during the project. Linear mixed models for repeated measures were used to analyze each daily activity (ie, sleep, outing activities, periods of low mobility, cooking-related activities, hygiene-related activities). Significant changes observed in data from monitoring reports were compared with information gathered by the care professional to explore concurrent validity.

**Results:**

Over time, the monitoring reports showed evolving trends in the care recipient’s daily activities. Significant activity changes occurred over time regarding sleep, outings, cooking, mobility, and hygiene-related activities. Although the nurse observed some trends, the monitoring reports highlighted information that the nurse had not yet identified. Most trends detected in the monitoring reports were consistent with the clinical information gathered by the nurse. In addition, the AAL system detected changes in daily trends following an intervention specific to meal preparation.

**Conclusions:**

Overall, trends identified by AAL monitoring are consistent with clinical reports. They help answer the nurse’s questions and help the nurse develop interventions to maintain the care recipient at home. These findings suggest the vast potential of AAL technologies to support health care services and aging in place by providing valid and clinically relevant information over time regarding activities of daily living. Such data are essential when other sources yield incomplete information for decision making.

## Introduction

### Background

Neurocognitive disorders affect 50 million individuals globally, with nearly 10 million new cases diagnosed each year [1]. Alzheimer disease (AD), the most common cause of dementia, is a disabling condition that has detrimental effects on memory, thinking abilities, behavior, and the ability to perform daily activities. By 2050, it is expected that one new case of AD will develop every 33 seconds, resulting in nearly 1 million new cases per year [2]. These numbers translate into an important global economic burden. Although the cost of providing medical and social care to individuals with dementia differs by country, the annual global cost of dementia in 2019 was estimated at US $1 trillion and is expected to double by 2030 [1]. As such, the World Health Organization recognizes it as a public health priority [3].

From the perspective of individual functional status, the speed and magnitude of decline varies, but the functional loss continuum begins with difficulties in completing complex instrumental tasks and continues until there is a complete loss of the ability to perform basic activities of daily living [4]. Instrumental tasks, including cooking, housekeeping, taking public transportation, and managing medication and finances, start declining in mild to moderate stages of the disease [4]. Basic activities of daily living—self-care activities such as eating, hygiene, grooming, and dressing—start declining in the moderate to severe stages of AD [4]. As such, throughout the progression of AD, older adults can become vulnerable to self-neglect.

Self-neglect is a behavioral condition that is characterized by the inability to sustain primary personal needs, including sufficient intake of food, personal hygiene, taking medication, and living safely [5]. Self-neglect predisposes older adults to devastating consequences such as multiple emergency department visits, maltreatment, nutritional deficiencies, nonadherence to medical treatment, and higher rates of morbidity, mortality, and nursing home placement [6-11]. The prevalence of self-neglect in older adults is high, ranging from 5% to 21%, on the basis of several factors [9], with the presence of cognitive impairments being the most important factor [12,13] even when the decline is mild [14-17]. The resulting decrease in independence in daily living has a significant impact on the individual’s ability to stay at home, which causes an increased risk of institutionalization [18,19]. However, research has shown that older adults with neurological impairments prefer to live independently in their own homes even if they may be dependent on others for managing their daily lives [20]. Moreover, recent research has found that, even with cognitive impairment, living at home is more beneficial to older adults than relocating to a nursing home. For example, it was shown that older adults living at home experience a better quality of life, have better cognitive function, are less depressed, and are more socially active, with effects remaining even after stratifying for severity of dementia [21-23].

### Ambient Assisted Living

Considering the growth of the aging population, the unprecedented economic pressures on the health and social care system this entails, and the benefits of staying at home for older adults, there is an urgent need to provide sufficient home care support to individuals with cognitive disorders.

In recent years, the use of ambient assisted living (AAL) systems has emerged as a way of promoting and extending aging in place. Implementing AAL systems involves deploying technologies (eg, sensors and actuators) in a home environment for the purpose of collecting continuous and real-time monitoring information about the environment (eg, temperature, humidity, and smoke in the home), the occupants (activities of daily living routines), and their health (eg, heart rate, body temperature, blood pressure, and blood oxygen level) [24]. AAL monitoring has many uses, such as detecting the occurrence of unusual or hazardous events (eg, a fall, cardiac arrest, or bradycardia) and alerting a dedicated resource person to provide immediate support and prevent dangerous situations. Another approach uses rich and reliable data from AAL monitoring to support clinical decision making regarding a care recipient’s state [25-27]. For example, home care professionals have used home monitoring to adjust their care plan on the basis of clinical and home monitoring data [27]. For instance, they could add interventions if through monitoring they observe that a care recipient is sleeping too much or skipping meals. In addition, in the context of applying technological solutions in the care of seniors with AD, Kaye [28] proposed that AAL monitoring may be used to capture meaningful real-time changes in individuals’ dementia trajectories and therefore plan preventive strategies. Considering the current limitations of functional assessments and the challenges associated with AD, Kaye suggested that AAL monitoring be used to improve health maintenance by offering “the opportunity to not only observe change in the person’s usual environment but also to more frequently, and in some cases continuously, monitor a subject for salient change” [28]. Indeed, this could facilitate the development of timely strategies to maintain independence through later life or predict progression to disability.

Although novel, AAL monitoring has already shown promising outcomes. A recent literature review [26] concluded that the most promising technologies are those that monitor activities of daily living and detect falls and changes in health status. In 2016, a comprehensive review of the frameworks and sensors used in various AAL systems also showed that such technologies are often used to assess immediate safety risks to promote older adults living independently by alerting someone when a worrying situation arises [29]. However, these authors found that AAL systems are not often used for analytics and decision making, particularly for long-term care. For example, environmental factors such as temperature, humidity, motion, light, and contact could be analyzed to monitor health decline or the efficacy of an intervention.

Nevertheless, implementing AAL monitoring in real-life clinical settings presents several challenges. According to Peetom et al [26], to provide data effectively, it is essential that monitoring technology be based on algorithms that enable clinically relevant changes and situations to be detected without an overabundance of false alarms. Although AAL monitoring can predict cognitive decline among large cohorts of older adults [30], it remains to be shown whether it can support clinical decision making at an individual level (eg, for a care recipient). Clinicians have raised concerns about technology overloading them with information, especially irrelevant information [25], and generally clinicians have little office time to examine lengthy reports. Thus, it is important for them to be able to visualize collected data in a way that is intuitive and relevant for clinical decision making [31]. Other studies have used machine learning approaches to predict conditions such as cognitive decline [32-36]. For example, Dawadi et al [33] used statistical features (variances, autocorrelation, skewness, kurtosis, and change) of daily activity behavior (ie, total sensor events, cook duration, sleep duration) to train machine learning algorithms to predict the clinical assessment scores. With it, they achieved an accuracy of 72% in classifying cognitive assessment scores. Although these are promising and effective approaches, the process to which scores are calculated based on machine learning are at times described as a black box, owing to their lack of transparency [37]. By lack of transparency, we mean that it can be difficult for a clinician to grasp *why* the system has reached a given conclusion and, afterward, to explain or justify how these scores influenced their decision making (ie, understanding *why* the system has reached a given conclusion can be unfathomable). Transparency is critical for commercial, legal, and clinical applications because professionals must justify their decisions on the basis of tangible observations [38]. This finding was supported in preliminary focus groups conducted within the context of this study, as care professionals explicitly stated that they did not wish to be provided a conclusion or told what to do; instead, they wished to receive valid information that could be used to enhance their decision making [27].

As such, in this study, we provided a care professional with AAL monitoring reports that showed transparent information on their care recipients’ daily routines (ie, time spent per day performing daily activities and standard deviation in time spent performing them). Statistical analyses applied to the collected data highlighted significant trends. This was done in a way that allowed the care professional to easily distinguish normal fluctuations from presumed significant changes in daily routines and to allow the care professional interpret these trends and take them into account in their decision-making process.

### Objectives

This study seeks to provide essential insights into the clinical relevance of AAL use in real-life settings to support the delivery of home care services over time. Specifically, we seek to examine the concurrent validity of AAL monitoring reports and the well-established methods used by care professionals to gather information, given that any discrepancy between these 2 methods would lessen the perceived value of monitoring reports. To achieve this goal, a single-case study design is used to compare the information gathered regarding one care recipient with AD. A single-case approach is recommended when changes over time need to be assessed repeatedly [39]. Moreover, it allows for a more in-depth examination of consistency between events that would be difficult to translate into group means, as is the case with AAL monitoring reports.

## Methods

This longitudinal single-case study is part of a larger project designed to understand how AAL monitoring can be successfully implemented in home care services and support the decision making of health and social care professionals in the public social and health care system [27]. The case in this study comprised one care recipient (*Lisette*) and the health and social care professional responsible for her home care support (referred to here as *the care professional*). Lisette’s assigned case manager is a clinical nurse with a Bachelor’s degree in nursing. She evaluates client health status and ensures that the nursing care and treatment plan is implemented for patients with complex health problems and provides care and treatment. As a case manager, her duties also include coordinating Lisette’s specific needs for care and services and supervising her home support [40].

### Recruitment

Health and social care professionals from the home care services of a local community health and social services center were invited to identify older adults who could benefit from AAL monitoring technology. To be included in the study, a care recipient had to be (1) receiving home care services due to a loss in functional autonomy related to a cognitive decline and (2) living alone. Care recipients were informed that AAL monitoring technology would be integrated into their home to monitor their daily routine (eg, sleeping and cooking). Care professionals were told that they would receive monthly reports on such activities to better understand the person’s daily functioning. They would also be interviewed to better understand how they found the experience of using the monitoring data reports. The project was approved by the Aging and Neuroimaging Ethics Review Board of the South-Central Montreal Integrated University Health and Social Services Center (CER VN 16-17-22). All participants signed an informed consent form before taking part in the data collection process. Lisette was identified as a fitting care recipient for the study by her care professional in January 2017.

### Data Collection

The AAL monitoring technology was installed in Lisette’s home on February 9, 2017. Her participation in the project ended a year and a half later, after her hospitalization and transfer to a long-term care facility, on July 6, 2018. Her data set was collected through a monitoring period that extended over 490 days, using 25 sensors and comprising approximately 617,000 logs. The sensors and algorithms used to create the monitoring reports are described later.

The monitoring reports were triangulated with multiple sources of data to examine their concurrent validity: medical file, verbatim of interviews with the care professional, emails, and memos of telephone exchanges (approximately 20) with the care professional. The care professional was interviewed on January 24, 2017 (before any monitoring report was sent); June 14, 2017; October 25, 2017; and October 26, 2018 (after Lisette’s hospitalization). These interviews were conducted by a researcher specializing in qualitative research (MC) and triangulated with the monitoring report outcomes [27]. The final interview took place 3 months after Lisette’s hospitalization due to uncertainty about Lisette’s transfer during that period.

### Case Description

Lisette was selected as a representative case for this study for several reasons. First, she was an older woman with AD who wished to remain in the apartment in which she had been living for several years for as long as possible. Second, the extensive data set collected through a 490-day monitoring period allowed for a rich analysis. Third, the monitoring began when the care professional became concerned about Lisette’s ability to live independently and continued until her transfer due to the deterioration of her condition. Therefore, we were confident that documenting this individual during this particular period would encompass significant changes in behavior, from beginning to end.

Lisette, a widowed older woman, was 91 years old at the time of her recruitment and had been living in the same one-bedroom apartment for the last 14 years. Her apartment was in a residence for independent seniors. Her son was her main caregiver and the primary contact for health care providers in case of an emergency. In 2015, she was diagnosed with AD, but a vascular etiology was also considered. Despite the diagnosis, she wished to continue living in her apartment, with assistance, and developed a social network with her neighbors. Although her son was considering whether Lisette should be transferred to a facility with a higher level of care, her daughter did not consider it necessary; thus, they had conflicting points of view about their mother’s level of independence. Their difference of opinion made it difficult for the care professional to gather reliable information to guide her own clinical decision making. Moreover, Lisette seemed to have limited awareness of her cognitive difficulties and their impact on daily living. Her son and the care professional mentioned that she was often unreliable when asked about her everyday routine and recent events.

In this context, Lisette’s care professional wished to acquire objective and reliable information regarding Lisette’s routine. This would enable her to better assess the safety risks related to maintaining Lisette in her home and to confirm or refute her clinical hypothesis before developing a comprehensive intervention plan. The main issues of concern identified by the care professional at the outset of the study were as follows: (1) malnutrition (because of food left untouched in the refrigerator over long periods of time and low body weight), (2) hazardous use of the stove (safety concerns expressed by the landlord), and (3) poor personal hygiene (slow shrinkage of the bar of soap observed by the care professional).

The monitoring period began on February 9, 2017, and ended on July 6, 2018. During this period, the care professional observed that Lisette’s AD symptoms progressed. During follow-up clinical visits, Lisette’s memory and orientation declined noticeably and continuously from December 2018 onward. Monitoring continued until Lisette experienced a serious episode of confusion and panic, calling her son because she was looking for her late husband. Following that episode, she was hospitalized on July 6, 2018, and shortly thereafter was moved by her family to a private home for older adults, ending the monitoring.

### Sensors and Algorithms

Three different types of sensors were integrated into the home environment: passive infrared (PIR) sensors (Everspring HSP02), magnetic contact sensors (Everspring HSM02), and smart electric switches (Aeotec ZW078 and ZW096). An attempt to integrate water sensors was unsuccessful due to rapid corrosion of the sensors when submerged in water and difficulties mounting them in a humid environment. Wireless sensors were used because they were easy to install in a real-life setting and could be moved to a new apartment if needed; these were considered important characteristics for decision makers in the context of a public health care system.

**Figure 1:**
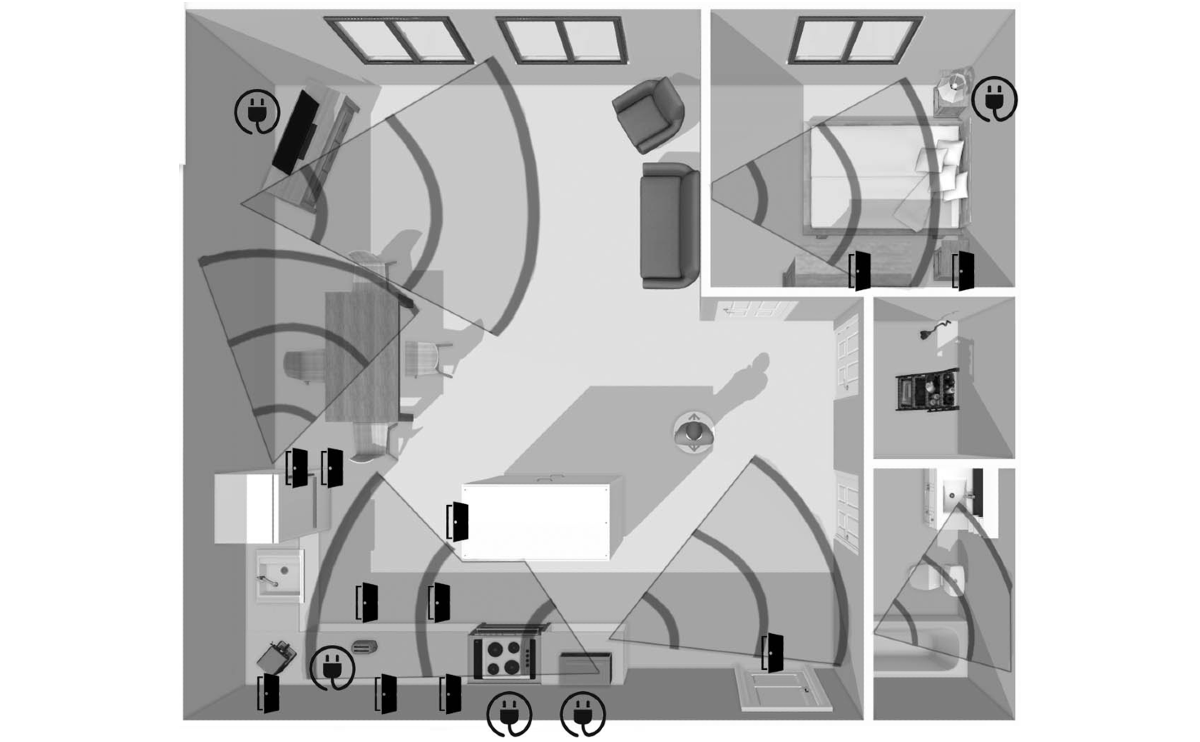
Sketch of the placement of sensors in Lisette’s apartment. Conic area: passive infrared motion sensors; door symbol: magnetic contact sensors; electric plug symbol: smart electric switches.

One PIR sensor per room was installed in the kitchen, dining room, and living room; at the entrance; and in the bathroom. Two were installed in the bedroom: one aimed toward the bed and another directed toward the door by which to exit the room. Electric sensors were connected to a television, a bed lamp, a microwave, a toaster, and a stove. Contact sensors were installed on the entrance door, on 2 dresser drawers in the bedroom, and on 2 food storage cabinets, the refrigerator, freezer, oven, utensil drawer, and 4 cupboards in the kitchen. Figure 1 illustrates a map of sensor deployment in Lisette’s apartment.

The selection and placement of wireless sensors were customized to specifically focus on daily activities that were relevant to Lisette’s care professional, namely, sleep habits (sleep), exiting the apartment (outing), periods of prolonged inactivity (low mobility), cooking-related activities (cooking), and hygiene-related activities performed in the bathroom (hygiene). Algorithms were built around various assumptions about these different activities, as previously described in a study by Lussier et al [27]. First, *room occupation* was recognized as follows: the occupant was considered to be in one room for as long as he or she was not detected in another room or outside the apartment. *Sleep* was identified if the occupant spent more than 20 min in the bedroom without interacting with any sensors other than the PIR directed at the bed. *Outing* was recognized if the following sequence of events occurred: (1) closing the entrance door, (2) no PIR activity in the home for more than 5 min, and (3) opening the entrance door. *Low mobility* was recognized if no PIR sensors and no contact sensors were triggered over a 15-min period and the occupant was not recognized as resting (in the bedroom) or being out. With respect to *cooking* activities (ie, meal preparation, washing the dishes, storing groceries), the assumption was that the occupant was cooking if several sensors placed in the kitchen were triggered over a short period. More precisely, for each 15-min period (ie, 3 PM to 3:15 PM, 3:01 PM to 3:16 PM, etc), a cooking score was established on the basis of the frequency and diversity of sensors triggered in the kitchen. If the score was 2 SDs higher than the average for that occupant, they were then recognized as cooking. This approach is similar to that used by Rantz et al [41] and was done to report only on activities that were significant in the context of the occupant’s daily routine. Finally, given that water sensors could not be successfully installed on the sink and bathtub faucets, the recognition of *hygiene* (ie, brushing teeth, showering, going to the toilet, washing hands) relied exclusively on prolonged presence in the bathroom. Similar to the cooking activity, a score was calculated on the basis of the number of minutes spent in the bathroom per 15-min window. The score then had to be 2 SDs higher than the average for that occupant to be recognized as engaging in a period of bathroom hygiene. It is important to note that it was impossible to verify whether the occupant or someone else (eg, the caregiver) was performing the actions. The care professional was aware of this limitation. See Textbox 1 for an overview of detailed algorithms.

**Overview of daily activity algorithms.**

##MAIN ALGORITHM

While(TRUE)

   Map(RoomName,Presence)=IndoorPIRMotionSensorList ()

   ROOMOCCUPATION=Map(RoomName,Presence)

   Sleeping=Event (Sleep(ROOMOCCUPATION))

   Outing=Event(Outings (ROOMOCCUPATION))

   LowMobility=Event(LMobility(ROOMOCCUPATION))

   Cooking=Event (Cook(ROOMOCCUPATION, standardDeviations(cookingScore)))

   Hygiene=Event (BathroomAct(ROOMOCCUPATION, standardDeviations(hygieneScore)))

End

MIN_OUTING_TIME=5 minutes

MIN_SLEEPING_TIME=20 minutes

MIN_LOWMOBILITY_TIME=20 minutes

##SUBFUNCTION ALGORITHM

##Sleep

IF(ROOMOCCUPATION in [Bedroom]>, MIN_SLEEPING_TIME)

   IndoorPIRMotionSensorLastTrigger([Bedhead] ,Duration))

##Outings

IF (ROOMOCCUPATION in [Entrance]]>MIN_OUTING_TIME)

ClosingEntranceDoor(True)

   IndoorPIRMotionSensorLastTrigger([],Duration)

##LMobility

IF(NOT(sleeping) & NOT(outing) & MagneticContactSensorLastTrigger>MIN_LOWMOBILITY_TIME & RoomOccupationLastChange>MIN_LOWMOBILITY_TIME)

IndoorPIRMotionSensorLastTrigger([] ,Duration))

##BathroomAct

IF(ROOMOCCUPATION in [Bathroom])

   BathroomSensorsUsed[IndoorPIRMotionSensorLastTrigger([Bathroom], MAX_ACTIVITY_TIME)]

   hygieneScore = FrequencyOfUse (BathroomSensorsUsed)

##Cook

IF(ROOMOCCUPATION in [Kitchen, Dining])

   KitchenSensorsUsed[IndoorPIRMotionSensorLastTrigger([Kitchen, DiningRoom], MAX_ACTIVITY_TIME)] +

   KitchenSensorsUsed[MagneticContactSensorLastTrigger ([Kitchen, DiningRoom], MAX_ACTIVITY_TIME)] +

   KitchenSensorsUsed[ElectricalMeasurementSensor ([Kitchen, DiningRoom], MAX_ACTIVITY_TIME))]

cookingScore = FrequencyOfUse(KitchenSensorsUsed)

### Monitoring Reports and Statistical Analysis

Each month postbaseline, the care professional was presented with a monitoring report sent via email. Reports were sent monthly because this was considered frequent enough by the care professionals. Monitoring reports were divided into 2 sections. The first section shows features from the previous month (eg, the time of day when daily activities were most likely to occur, a pie chart of the different room occupancy averages within the home; see Lussier et al [27] for an example). The second section, which is the main focus of this paper, shows the monthly evolution of the average time per day spent performing each of the 5 daily activities. It included the evolution of the frequency and average duration of stove and microwave use as well.

To determine which lifestyle changes should be highlighted as significant to the care professional, linear mixed models for repeated measures were used for each activity (ie, sleep, outing activities, periods of low mobility, cooking-related activities, and hygiene-related activities). Outcomes were expressed as the daily duration of time spent performing these activities. In Lisette’s case, 490 continuous days of monitoring were divided into 14 months. Although the term *month* will be used for the sake of simplicity, 35-day periods were used instead of calendar months. This was done so that each month contained the same number of days, with 5 occurrences of each day of the week (ie, Monday to Sunday). This further allowed us to control for any discrepancy in weekly routines (eg, having a dance course every Sunday afternoon). Fixed factors were defined as months and weekdays. The covariance structure selected was compound symmetry for all the tested responses and was determined according to the Akaike information criterion. For outcome, *the marginal means of each month were compared with the initial baseline month. P* values were not adjusted for multiple comparisons as each observation was compared with the baseline observation. This was also done to preserve the statistical power of this exploratory experiment, as we did 13 comparisons, adjusting the *P* value accordingly was considered too restrictive. All analyses were conducted with an *α* threshold of .05, using IBM SPSS Statistics version 25.

For each activity, 2 sets of analyses were performed. First, we examined statistically significant overall trends, as a trend could correlate with cognitive or health decline. Second, we compared the baseline report (ie, the first month of monitoring) with each following month to determine if, and which, months significantly differed from the baseline. On the one hand, if a general trend was observed, this could be used to help determine at which month a trend reached significance. On the other hand, this could be used to identify irregular months that did not correspond to any overall trend. This could further be useful to pinpoint important but nonpermanent changes in the daily routine for the care professional to explore. It is also important to note that monitoring reports were part of an ongoing process; therefore, what could initially appear to be an irregular month could in fact become part of an ongoing trend as months passed by.

### Concurrent Validity

To examine the concurrent validity of AAL monitoring reports for each of the main activities monitored (sleep, outing, low activity, cooking activities, and hygiene), we first used statistical analyses and values for changes in daily activities that were highlighted in the monitoring reports. We then explored whether any of the significant changes from the monitoring data could be linked with information gathered by the care professional. This information was extracted from interviews, emails, and memos exchanged with the care professional and information from the care recipient’s medical file.

We assumed that significant changes calculated from the monitoring data would be coherent with real-life information, thereby validating the potential of this approach in a clinical setting. Moreover, because the care professional may lack continuous and reliable information, we expected that monitoring reports would detect changes in activities of daily living that the care professional would not have been aware of.

## Results

For each of the main activities monitored (sleep, outing, low activity, cooking activities, and hygiene), the results are provided in 2 sections. The first section presents statistical analyses and values for significant changes in the monitoring reports (see Table 1 for means and 95% CI of repeated measurements). The second section compares each significant change with information gathered by the care professional to explore concurrent validity. For each value, the mean and SD are presented.

**Table 1:** Means and 95% CI of repeated measurements for sleeping outings, cooking activities, hygiene, and low mobility during the monitoring period.

Month	Sleep, mean (95% CI)	Outings, mean (95% CI)	Cooking activities, mean (95% CI)	Bathroom usage, mean (95% CI)	Low mobility, mean (95% CI)
1	8.36 (7.93 to 8.80)	3.11 (2.49 to 3.72)	1.50 (1.28 to 1.72)	1.96 (1.71 to 2.20)	3.96 (3.18 to 4.74)
2	8.53 (8.09 to 8.97)	3.65 (3.04 to 4.27)	1.24 (1.02 to 1.46)	2.22 (1.92 to 2.53)	3.88 (3.10 to 4.66)
3	8.66 (8.24 to 9.10)	3.15 (2.54 to 3.77)	1.17 (0.95 to 1.39)^a^	2.07 (1.79 to 2.35)	3.74 (2.96 to 4.52)
4	9.08 (8.60 to 9.57)^a^	2.73 (2.06 to 3.41)	1.02 (0.78 to 1.27)^a^	1.66 (1.39 to 1.93)	3.93 (3.06 to 4.80)
5	8.71 (8.27 to 9.15)	2.54 (1.93 to 3.16)	0.77 (0.55 to 0.99)^a^	1.66 (1.41 to 1.90)	4.12 (3.31 to 4.92)
6	9.37 (8.88 to 9.85)^a^	1.22 (0.60 to 1.85)^a^	0.73 (0.49 to 0.97)^a^	1.76 (1.51 to 2.01)	4.63 (3.76 to 5.51)
7	8.96 (8.48 to 9.43)	1.40 (0.76 to 2.05)^a^	0.80 (0.57 to 1.02)^a^	1.65 (1.40 to 1.91)	5.94 (5.11 to 6.77)^a^
8	9.42 (8.96 to 9.88)^a^	1.40 (0.78 to 2.01)^a^	1.10 (0.88 to 1.32)^a^	1.84 (1.60 to 2.09)	4.08 (3.25 to 4.91)
9	9.90 (9.42 to 10.39)^a^	0.57 (−0.06 to 1.21)^a^	1.03 (0.80 to 1.26)^a^	1.87 (1.62 to 2.13)	5.38 (4.55 to 6.21)^a^
10	9.77 (9.30 to 10.24)^a^	1.56 (0.94 to 2.18)^a^	1.14 (0.91 to 1.36)^a^	1.47 (1.22 to 1.72)^a^	4.21 (3.37 to 5.06)
11	9.74 (9.30 to 10.19)^a^	2.05 (1.44 to 2.67)^a^	1.71 (1.49 to 1.93)	1.62 (1.37 to 1.86)	3.83 (3.03 to 4.62)
12	9.72 (9.24 to 10.20)^a^	1.85 (1.24 to 2.47)^a^	1.76 (1.54 to 1.98)	1.72 (1.47 to 1.96)	4.84 (4.06 to 5.62)
13	10.11 (9.65 to 10.57)^a^	1.17 (0.53 to 1.80)^a^	1.23 (1.00 to 1.46)	1.56 (1.29 to 1.83)^a^	4.29 (3.49 to 5.10)
14	10.18 (9.74 to 10.62)^a^	0.95 (0.33 to 1.56)^a^	1.05 (0.83 to 1.27)^a^	1.36 (1.12 to 1.61)^a^	3.43 (2.64 to 4.21)

^a^Statistically significant (*P*<.05) changes compared with the first period.

### Sleep Habits

#### Monitoring Report

**Figure 2:**
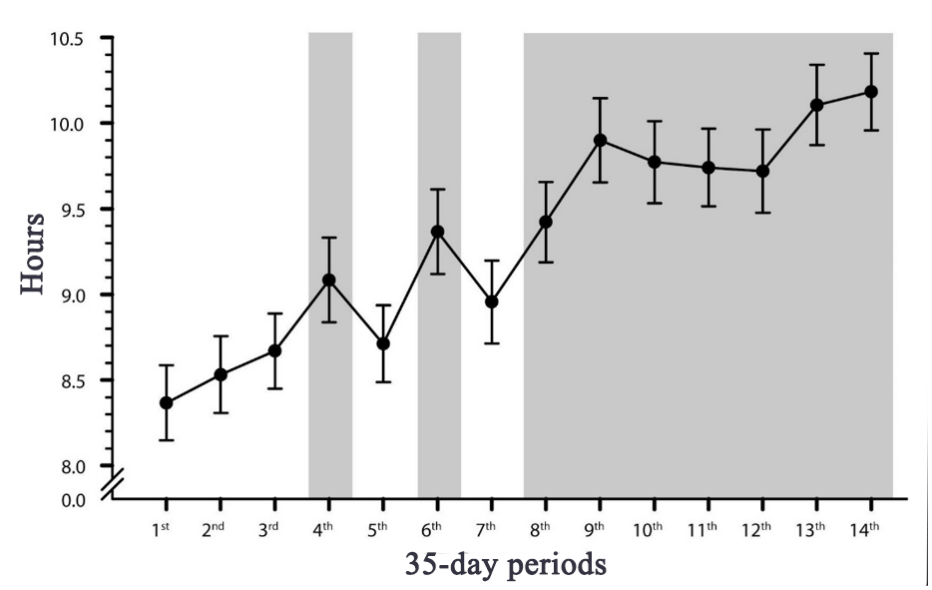
Evolution of estimated means (SE) for hours of sleep detected during the monitoring period. The gray zones highlight statistically significant (*P*<.05) periods when compared with the first period.

Over the course of monitoring, Lisette gradually spent more time resting (month effect: *F*_13, 397.98_=7.05; *P*<.001). This trend became steady and significant from the eighth month onward. In the last month, sleep was detected for 10.18 (SD 1.64) hours, which represents a 22% increase in resting time when compared with the baseline (8.37, SD 1.27 hours; Figure 2). In addition, the monitoring data suggested that she more frequently woke up later in the morning toward the end of the monitoring period. The data showed that she woke up between 7:25 AM and 7:56 AM during the first month but between 7:33 AM and 10:30 AM over the last month of monitoring. There were no indications that she woke up more frequently during the night.

#### Clinical Observation

Interestingly, Lisette did not mention to the clinician that she was feeling more tired or sleeping longer than before. However, the personal care assistant responsible for giving her medication each morning (a different health professional, not the one in charge of coordination and treatment plan) noted that she had to wake her up more often when visiting.

### Outings

#### Monitoring Report

**Figure 3:**
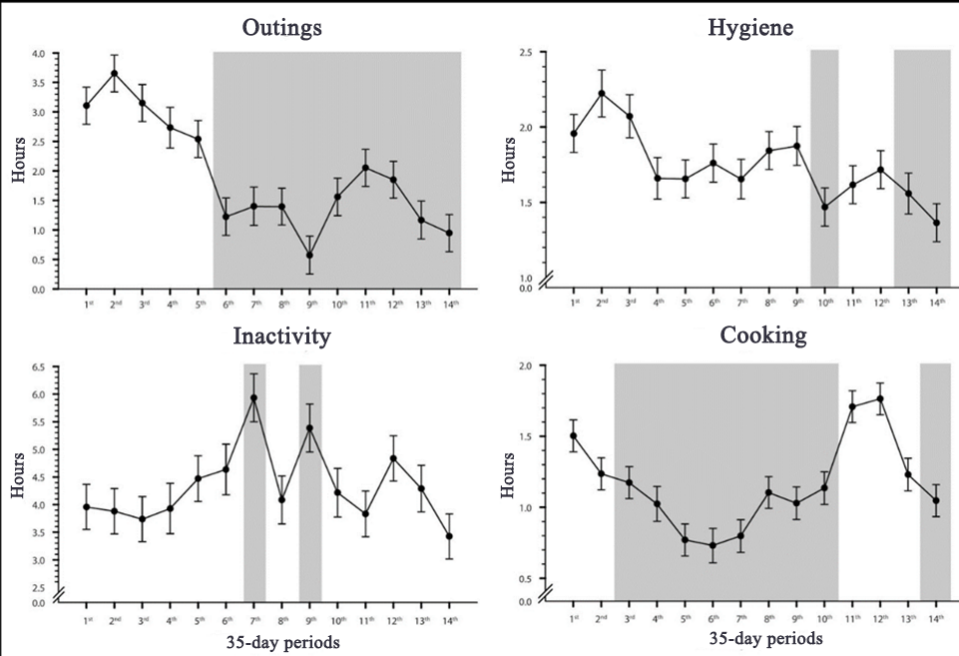
Evolution of estimated means (SE) for outings, bathroom usage, low mobility, and cooking activities during the monitoring period. The gray zones highlight statistically significant (*P*<.05) periods when compared with the first period.

Overall, Lisette gradually went outside for shorter periods (month effect: *F*_13, 443.00_=8.70; *P*<.001; see Figure 3, top-left). This change became steady and significant from the sixth month onward. The reports highlight that Lisette spent 68% less time outside when compared with the baseline (3.10, SD 2.80 hours) and to the last month (0.97, SD 0.90 hours). Another noticeable decline in outings was observed specifically in December (0.35, SD 0.23 hours).

#### Clinical Observation

Trends in outings were corroborated by Lisette, who mentioned to the care professional that she had lost interest in the social activities held at her building because she said she disliked the new hosts in charge of the activities. She also mentioned that she had stopped attending the dance activities on weekends because she did not appreciate the change in the style of music played. There was no way for the clinician to verify that information. As for the decline observed for the month of December, it was noted in her medical record that Lisette had influenza in December and was quite incapacitated by it, which would most likely account for her staying at home.

### Cooking Activities

#### Monitoring Report

Over time, a significant decline in cooking-related activities was observed (month effect: *F*_13, 424.81_=7.83; *P*<.001; see Figure 3, top-right). However, the time spent on these activities did not follow a steady decline. A first significant decline was observed in the third month, and the decline continued gradually up to the sixth month, the lowest point reached (42.24, SD 26.40 m), representing a 53% decline when compared with the baseline (90.00, SD 50.00 m). Cooking activities slowly increased over the 11th to 13th month, reaching a value comparable with that of the first month. Finally, cooking activities started decreasing again during the last month (62.40, SD 30.61 m), a 31% decline compared with the first month.

The stove was used on about 16% of days during the monitoring period (the oven with or without the burners, 13%, and only the burners, 3%). On days the stove was used, burners were used for an average of 10 (SD 11) min and the oven for 15 (SD 10) min. Only one instance of dangerous stove use was identified: Lisette left the apartment while the stove was switched on and then came back but only turned it off the next morning. The microwave was used on 32% of days for an average of 6 (SD 6) min per day. On one occasion, the microwave was in use for 20 min nonstop, which was a clear outlier for Lisette. Other than that, no unusual behaviors were detected.

#### Clinical Observation

The patterns related to cooking activities were supported by real-life information. Notably, when the care professional received monthly reports illustrating raw data, she noticed a change in pattern. Considering her concern that Lisette was at risk of malnutrition, she used that information to discuss Lisette’s eating habits with her son during the summer (exact date unknown, but between the fifth and seventh month). This conversation led to a change in the caregiver’s behaviors: now more aware that his mother preferred homemade meals to ready or frozen meals, and to increase meal intake, the son said he would prepare more homemade meals for her. He and his wife would also come more often to cook for her and put leftovers in the refrigerator. This decision correlated with increased detection of cooking-related activities in Lisette’s home for several months after this exchange. Therefore, the increase in time spent cooking at that time is a result of the compensatory behaviors by family members (ie, family cooking at Lisette’s home and Lisette eating more regularly because the food she liked was easily available). However, this compensatory behavior either did not last or was not sufficient support, as Lisette’s cooking-related activities again declined in the last 2 months before her hospitalization and the end of monitoring.

Overall, the infrequent use of the microwave and stove was consistent with the care professional’s suspicions that Lisette might not be eating hot meals daily (as indicated by the accumulation of prepared meals in the refrigerator). Concerning the dangerous use of the stove, a member of the research team contacted Lisette shortly thereafter to verify if the sensor was defective. She said she forgot the burner on and burned her fudge during the night. The care professional was informed of the event. As for the microwave, the care professional questioned Lisette about this incident a couple of weeks later. She answered that she probably meant to set 2 min but added a *0*, resulting in 20 min instead.

### Hygiene

#### Monitoring Report

The monitoring results suggest that hygiene-related activities declined significantly over time (month effect: *F*_13, 40658_=2.94; *P*<.001), reaching a 30% decline in time spent in the bathroom when comparing the baseline (117.36, SD 56.80 m) with the last month (82.98, SD 25.80 m; see Figure 3, bottom-left). This trend first became significant during the 10th month but remained steadily significant only from the 13th month onward. Activation of the clothing drawers’ sensors remained stable over time.

#### Clinical Observation

As changes in hygiene were detected late in the study, the care professional did not have many opportunities to gather information on this aspect before Lisette was hospitalized and transferred. However, she mentioned that a decline in hygiene would not be surprising, considering the rapid decline of her cognitive state in the month preceding her transfer. Moreover, post hoc analyses suggest that the longest bathroom hygiene periods tended to occur before outings. Therefore, a decline in hygiene-related activities would be consistent with Lisette’s confirmed abandonment of some social activities.

### Low Mobility

#### Monitoring Report

The average period of low mobility remained quite stable over time (month effect: nonsignificant.; see Figure 3, bottom-right). The only 2 significant increases in periods of low mobility occurred in the seventh month (5.27, SD 3.21 hours; *P*<.001) and the ninth month (4.69, SD 1.65 hours; *P*=.02) compared with baseline (3.96, SD 1.98 hours).

#### Clinical Observation

Although there was no clear explanation available for the increase in inactivity in the seventh month, Lisette’s medical record reported that she was sick with influenza during the ninth month, which could explain the fewer outings and more inactivity being detected in the home.

## Discussion

### Principal Findings

This study describes a longitudinal single-case study in a Canadian public home care setting. The objective was to examine the concurrent validity of AAL monitoring reports and care professional descriptions of real-life changes in activities of daily living experienced by an older adult diagnosed with AD (ie, Lisette). Lisette’s care professional received monthly monitoring reports of sleep, low mobility, outings, cooking, and hygiene-related activities. In the monitoring reports, algorithms and linear mixed models for repeated measures were used to highlight significant changes in Lisette’s daily activities. Highlights from the reports were then juxtaposed to information gathered by the care professional to determine if they concurred. Lisette's health and life events were gathered from her medical file and from interviews, emails, and telephone exchanges with her care professional.

As expected, statistically significant changes in daily routine were detected over the 490 days of monitoring. Through interviews conducted with the care professional, it was possible to conclude that monitoring report trends were consistent with the clinical information collected when staff visited the care recipient. In fact, a priori interrogations and observations made by the care professional were indeed reflected in the monitoring reports. For instance, the care professionals believed that Lisette was not cooking complex meals, and this was later confirmed by the monitoring report. In addition, the outcome of subsequent interventions was observable in the monitoring report. This occurred when the care professional invited the family to participate more in meal preparation, which led to a detectable change in routine. Interestingly, in addition to validating the initial hypotheses, the monitoring reports drew attention to certain unforeseen or unexpected changes that were then triangulated with other information gathered by the clinician. On occasion, this led to discussions with the care recipient. For example, when there was a temporary decline in outings and an increase in low-mobility periods during the month of December, the care professional asked Lisette about it. She then learned that Lisette had caught the flu that month, which was supported by information in her medical file. Another example is the gradual increase in the time spent sleeping, which Lisette was not aware of (or denied) but was corroborated by a personal care assistant. As such, by combining information from the monitoring report with comments made by another care worker, the care professional was able to gain a better understanding of Lisette’s situation that would have otherwise been overlooked, unattainable, or ambiguous.

### Comparison With Prior Work

This study is innovative because monitoring reports were designed in collaboration with care professionals to specifically address their requirements and the care professional’s need for information that would enable the best client support possible were carefully examined. Moreover, this technology was implemented in concert with the head of service to be included harmoniously with actual current services from the public health care system. Importantly, the evolution of daily routine observed in this case study was highly consistent with the current literature on daily activities in AD. For instance, sleep disturbance is prevalent and predictive of cognitive decline in older adults and in those with neurocognitive disorders [42]. Three studies using AAL monitoring technology to monitor sleep found that sleep quality and sleep hygiene measures were related to mild cognitive disorders in older adults [43-45]. More precisely, it is suggested that AD is not associated with more sleeping time but with less sleep efficiency (ie, lower percentage of time in bed spent asleep) [46]. With the sensors used in this case study, it was not possible to distinguish the time spent sleeping from the time spent lying down in bed. Nonetheless, although Lisette did not report any change in her sleep routine when asked, it is possible to assume that her sleep quality decreased because she needed to spend more time lying down in her bedroom as the disease progressed. The decrease in time spent outside the home was partially explained by Lisette, who mentioned withdrawing from social activities. She justified this behavior by referring to different changes in the way the activities were held. However, this behavioral evolution is also consistent with several studies showing that older adults with cognitive impairment reduce their engagement in social activities as their cognition declines [47-50]. Anosognosia (ie, a lack of awareness of deficit) is often observed early in the course of AD [51], so it is possible that Lisette withdrew from social activities because of cognitive decline but without being aware of that change. It is also at this stage that agitation, confusion, and distress episodes, such as the one Lisette experienced just before being moved out of her apartment, occur more frequently [52]. Therefore, anxiety and distress might have kept her from going out. Finally, the decline in hygiene occurring last in the timeline is also consistent with the literature. Indeed, while a decline in the ability to perform basic activities of daily life is minimal in the early stages of AD, moderate stages bring incipient difficulties with this sphere [53]. For example, at this stage, the care recipient should be able to shower alone but may forget to do so regularly. Therefore, the decline in hygiene was consistent within the context of progressing dementia and could be considered an indicator of worsening symptoms.

In comparison with other similar studies on AAL and clinical reasoning, our study is also in line with the study by Rantz et al [41]. In their study, Rantz et al [41] showed that monitoring the increase in activity level in the bathroom could support the detection of early signs of urinary tract infection by a nurse. To our knowledge, our study is however the first to document the use of daily living monitoring over a long period, in relation to other clinical data to support the clinical reasoning of health care providers. Monitoring data related to activities of daily living have great potential to support the work of home care providers via telehealth modalities. Faced with unprecedented challenges in allocating resources, in urban and remote rural areas, vast countries such as Canada could use AAL tools to better allocate services to the right person, at the right moment, ensuring more equitable and sustainable use of public health care capacities [54].

### Limitations

Although the results of this case study are promising, further replications among care recipients with similar medical conditions and in a variety of environments are necessary. The limitations of the monitoring technology used must also be addressed. First, it was impossible to accurately determine whether more than one occupant was in the home at the same time. Therefore, visitors’ activities were averaged in the data reports. The multioccupant dilemma must be further explored to develop a simple but accurate solution. This solution should not require wearable sensors, as poor compliance has been reported with this technique in older adults with cognitive deficits [55]. Nevertheless, this study focused on the question, “Has the activity been done?” rather than “Who is doing the activity?” because our participant was living alone. From the perspective of the care professional using the monitoring reports to decide if any additional services are needed or not, the main interest is in knowing that the activity is being carried out regularly, regardless of who has done it; a decision taking into account the support given by the caregivers, not in abstraction of it. Furthermore, care professionals are particularly interested in AAL monitoring technology for care recipients who live alone with minimal caregiver support as social isolation is a major risk factor for security and reliable sources of information are often lacking in this context [27]. Finally, monitoring reports are not a substitute for a functional, performance-based evaluation and should not be used.

### Conclusions

Although living at home presents several benefits for older adults [4,22,23], dealing with the increase in the incidence of self-neglect also poses great challenges for the health care system. This increases the demand for better resource allocation and innovative strategies to attenuate the estimated impact on health care expenditures. With the technology boom of the past decades, AAL monitoring systems are among the most promising tools to support the outcomes of individuals whose cognitive declines limit their effective participation in daily activities. AAL may improve the ability of older adults to cope at home and handle tasks and needs, which are the basis of the well-known aging-in-place design for living. Moreover, global efforts to cope with the COVID-19 pandemic of 2020 have acutely highlighted that it is crucial to have a system of health and social services that allow for remote monitoring of fragile older adults living alone and isolated under conditions of social distancing [56].

In this paper, we showed how an AAL monitoring system, using customized wireless sensors, was relevant during the assessment of home care services for an older woman with AD at risk of self-neglect. We were able to monitor the care recipient’s routine of basic (hygiene, sleep) and instrumental (cooking, outing) activities of daily living. We showed that nonintrusive AAL monitoring can identify and present the relevant trends in daily routines in data format. This continuously gathered information can then be integrated with other sources of information to help care professionals manage risks and develop tailored intervention plans.

Replication of this study will be required to strengthen the findings of this study. Future studies also need to consider the economic benefits of accessible AAL monitoring for clinical decision making in public and private health services. Such efforts are driven by a pressing need to deliver efficient home care services, enhance the quality of life of home care recipients, and reduce the burden on informal caregivers. We believe these results show that with further development and broader implementation, AAL monitoring systems will become essential tools in the promotion of aging in place.

## Abbreviations

AAL: ambient assisted living
AD: Alzheimer disease
FRQS: Fonds de la recherche du Québec—Santé
PIR: passive infrared
